# Vimentin Protein In Situ Expression Predicts Less Tumor Metastasis and Overall Better Survival of Endometrial Carcinoma

**DOI:** 10.1155/2022/5240046

**Published:** 2022-03-14

**Authors:** Xuefang Zhang, Guangming Cao, Xiaoli Diao, Wenyu Bai, Yang Zhang, Shuzhen Wang

**Affiliations:** ^1^Department of Obstetrics and Gynecology, Beijing Chaoyang Hospital, Capital Medical University, Beijing, China; ^2^Department of Pathology, Beijing Chaoyang Hospital, Capital Medical University, Beijing, China; ^3^Department of Obstetrics and Gynecology, Fangshan First Hospital, District, Beijing, China; ^4^Department of Anesthesiology, Beijing Chaoyang Hospital, Capital Medical University, Beijing, China

## Abstract

**Background:**

Vimentin, a cytoplasmic intermediate filament protein, has been recently identified to be a prognostic biomarker in some cancers. However, the function of vimentin in endometrial carcinoma (EC) remains unclear. Our study aimed at evaluating vimentin expression in EC and preliminarily exploring the role of vimentin in EC progression.

**Methods:**

In total, 341 EC patients who underwent surgical follow-up were enrolled in the retrospective study. Vimentin expression levels in EC tissues were analyzed using immunohistochemistry. Furthermore, the vimentin (VIM) gene expression levels in 547 samples in The Cancer Genome Atlas (TCGA) were analyzed. To examine the prognostic value of vimentin in EC, Kaplan-Meier survival analysis was performed, and a Cox model was established. Gene set enrichment analysis (GSEA) was also conducted using the Kyoto Encyclopedia of Genes and Genomes (KEGG) database to explore the role of vimentin in EC progression.

**Results:**

Negative vimentin expression in EC correlated significantly with lymph node metastasis, deep myometrium invasion (MI), lymph vascular space invasion (LVSI), advanced Federation International of Gynecology and Obstetrics Association (FIGO) stages (III and IV), and high tumor grade. Vimentin negativity was more common in type 2 EC than that in type 1 EC, and vimentin-negative patients had poorer overall survival compared with vimentin-positive patients. The results of GSEA suggested that vimentin may interact with classical pathways in EC.

**Conclusions:**

Negative vimentin expression correlates with tumor metastasis and worse overall survival in EC, suggesting that it may be an excellent prognostic biomarker for this disease. The mechanism by which vimentin contributes to EC progression needs to be explored in the future.

## 1. Introduction

Endometrial carcinoma appears to be increasing in incidence and mortality. It is the most frequent gynecologic malignancy worldwide [1]. Indeed, 417,367 new cases and 97,370 deaths due to corpus uteri cancers were estimated in 2020, making uterine cancer accounting for 4.5 percent of the 9 million new cancers in women in the world [2]. EC is classified into endometrioid (type 1) and nonendometrioid (type 2) subtypes based on histology [3]. A poor prognosis is common for type 2 EC; some early type 1 EC cases may be cured but relapse quickly after the initial therapy [4]. Therefore, it is not sufficient or appropriate to develop therapies that depend on usually used risk factors, such as surgical pathological stage, depth of myometrium invasion, or age. In 2013, The Cancer Genome Atlas indicated that it is possible to conduct stratified treatment according to biomolecular typing [5]. However, the mortality rate of EC has not decreased, despite novel pathogenetic and molecular discoveries, and the high-cost of complex molecular sequencing technology is an obstacle to its application in developing countries. Hence, novel immunohistochemical markers need to be explored to solve this problem.

Vimentin is a cytoplasmic intermediate filament protein considered to act as a marker of mesodermal origin [6]. In addition to being expressed in mesenchymal cells, vimentin plays an important role in the epithelial-mesenchymal transition (EMT), though its functional contribution to that process remains unclear [7, 8]. Overall, EMT is important for tumorigenesis in various cancers, which makes epithelial cells lose their polarity, decrease adhesion among cell-to-cell and cell-to-extracellular matrix, and increase the invasiveness of tumor cells [6, 9, 10]. Overexpression of vimentin correlates significantly with poor prognosis in several cancers, such as gastric cancer and breast cancer [11, 12]. Conversely, previous studies showed that vimentin immunoreactivity was common in normal proliferative endometrium, and its persistence in EC might indicate a less malignant phenotype [13–15]. For example, Papadopoulos et al. [14] demonstrated that expression of vimentin decreased as a lesion progressed to malignancy, and a recent study by Nesina et al. [15] also found that decreased expression of vimentin in EC correlated with the aggressiveness of tumors. However, there are only a few studies thus far focusing on the relationship between vimentin expression and EC prognosis currently, and its underlying molecular mechanism in EC is unclear. Furthermore, our study is the first study with such a large cohort and will serve as a basis for future studies.

Overall, vimentin expression and function differ in various types of cancer, and vimentin as a potential molecular target for the treatment of cancer has been proposed [16]. But the relationship between vimentin and EC is still not confirmed. Here, we reported a large cohort study to evaluate the possibility of vimentin expression as a prognostic marker in EC using immunohistochemical analysis, and TCGA database was used to verify our results. Also, we aimed to preliminarily explore the role of vimentin in EC progression by gene set enrichment analysis.

## 2. Materials and Methods

### 2.1. Patient Characteristics

**Figure 1:**
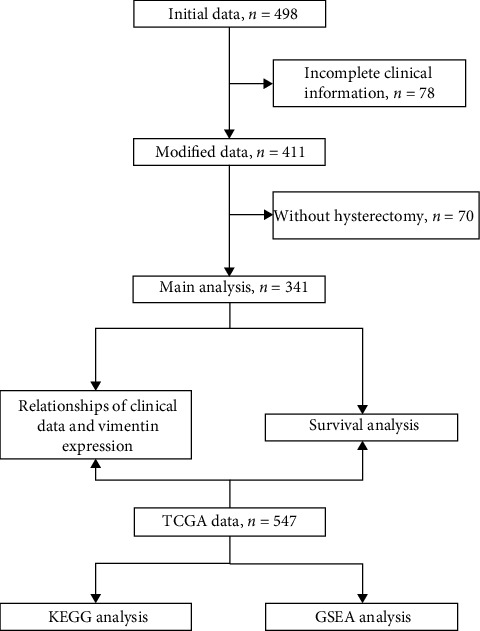
Flow chart of the study.

A total of 341 EC patients recorded in the Gynecology Department of Chaoyang Hospital between January 2012 and July 2021 were enrolled in this study. The study was conducted in accordance with the Declaration of Helsinki (as revised in 2013) and was approved by the Ethics Committee of Beijing Chaoyang Hospital (no. 2019-331). The procedure of the study was summarized in the flow chart (Figure 1).

Patients underwent total abdominal or radical hysterectomy plus bilateral salpingo-oophorectomy. The patients with incomplete clinical information, without hysterectomy or with other cancers concurrently were excluded. Lymph node sampling or dissection was performed in a total of 330 (96.8%) patients with tumors characterized by myometrial invasion and/or high-grade and/or uterine cervical invasion. Eleven cases in stage I did not undergo lymphadenectomy because magnetic resonance imaging (MRI) or computed tomography (CT) showed negative lymph node. High-risk patients underwent external radiotherapy or cisplatin-based chemotherapy after the primary surgery. After completing treatment, the patients were enrolled in routine surveillance programs.

Written informed consent was routinely required to collect clinical data and paraffin-embedded sections for research use. Histopathology was classified according to FIGO guidelines. Histologic tumor grading was assessed according to the FIGO grading system based on the ratio of glandular or papillary structures versus solid tumor growth (grade 1, <5% solid tumor; grade 2, 6-50% solid; and grade 3, >50% solid) [17]. Positive lymph nodes were defined that tumor cells can be found in the lymph nodes. All histologic sections were evaluated by two expert gynecologic pathologists, and those controversial cases were subjected to the diagnostic judgment of the other pathologists until a final agreement was achieved.

Demographic data and clinical information were collected from hospital records. The clinical and pathologic features of the 341 EC patients are summarized in Results and listed in Tables 1 and 2. The patients were followed up until September 2021 or death. The median follow-up time was 22 months (range 2~123). Information about vital status was obtained from medical records. Thirteen patients were lost to follow-up, and their data were censored in Kaplan-Meier analysis. Among the 13 patients, the histology for one case was clear cell carcinoma; the other 12 cases were type 1 EC. Overall survival (OS) was identified as the time from biopsy diagnosis to death.

### 2.2. Immunohistochemistry (IHC) Analysis

All specimens were embedded in paraffin; the blocks were cut as 4 *μ*m thick serial sections and baked dry. The streptavidin-biotinidase complex (SP) method was used, and all immunohistochemical procedures were carried out strictly in accordance with the kit instructions. The primary antibody against vimentin (Cell Signaling Technology) was diluted to 1 : 200 for use. Known positive slices were employed as the control, and phosphate buffered solution (PBS) solution was used as the blank control instead of the primary antibody [18].

**Figure 2:**
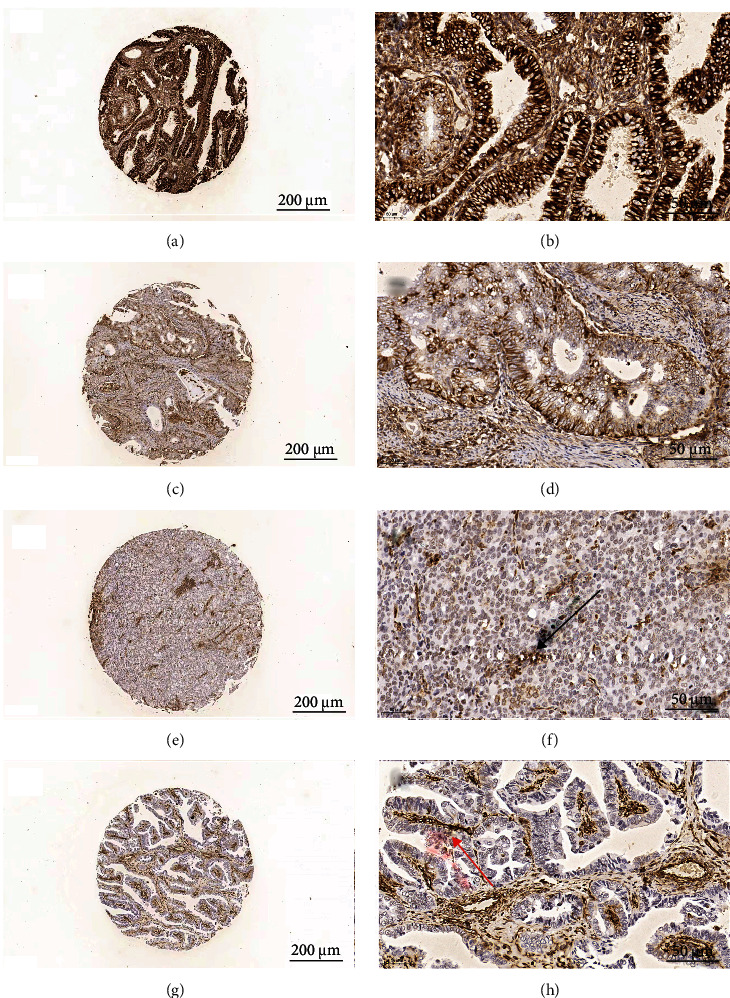
Immunohistochemical manifestations of positive vimentin and negative vimentin. (a, b) A highly differentiated endometrioid adenocarcinoma showed positive vimentin. Scale bar 200 *μ*m and scale bar 50 *μ*m. (c, d) A moderately differentiated endometrioid adenocarcinoma showed positive vimentin. Scale bar 200 *μ*m and scale bar 50 *μ*m. (e, f) A lowly differentiated endometrioid adenocarcinoma showed negative vimentin. Scale bar 200 *μ*m and scale bar 50 *μ*m. An arrow in black showed a blood vessel. (g, h) A serous papillary carcinoma showed negative vimentin. Scale bar 200 *μ*m and scale bar 50 *μ*m. An arrow in red showed tumor cells.

Vimentin expression was evaluated in samples with immunoreaction in a minimum of 500 histologically identified neoplastic cells. In the case of disagreement, the sections were subjected to joint evaluation using a multiheaded microscope. Expression of vimentin is mainly localized in the cytoplasm, with or without focal nuclear staining. Positive expression of vimentin was indicated by brownish yellow granules in the cytoplasm or nucleus (Figure 2). The intensity of vimentin staining was scored as follows: 0 (negative), 1 (weak), 2 (moderate), and 3 (strong). The percentage of positive cells was as follows: 0 (0–5%), 1 (6–25%), 2 (26–50%), 3 (51–75%), and 4 (76–100%). Negative and positive expression scores were calculated by multiplying the intensity score by the percentage of positive cells. For statistical analysis, the samples were classified into negative group (score ≤ 1) and positive group (score ≥ 2), as previously suggested [19].

### 2.3. VIM Gene Expression in TCGA

To validate our findings, EC patient samples (*n* = 547) from TCGA were used to correlate VIM gene expression with clinical outcome. Both clinical and gene expression data (RNA-Seq) were collected from Genomic Data Commons (GDC) (https://portal.gdc.cancer.gov/). Clinical data, including age, body mass index (BMI), menopause status, histologic grade, residual tumor, histological type, overall survival time, and vital status, are listed. Transcriptomics data were extracted as FPKM (fragments per kilobase of exon per million mapped reads) values for corresponding patients.

Patients were classified into two groups based on VIM FPKM value. Prognosis was examined by Kaplan-Meier survival estimators and compared using log-rank tests. To choose the optimal FPKM cutoff value for stratifying the patients into two different prognostic groups, the most significant *P* value within the 20th to 80th percentile was used, and the cutoff value for low and high VIM expression groups was set at 15.2 FPKM.

### 2.4. Gene Set Enrichment Analysis

We conducted GSEA to investigate the detailed molecular mechanisms of vimentin in EC. Gene expression data was downloaded from the official website of TCGA. GSEA (http://software.broadhttp://institute.org/gsea/index.jsp) was conducted using the Kyoto Encyclopedia of Genes and Genomes (KEGG) database with the GSEA v3.0 software. Enriched pathways correlating with VIM were detected by KEGG pathway enrichment analysis. A *P* value < 0.05 and an FDR (false discovery rate) < 0.25 were set as the cutoff.

### 2.5. Statistical Analysis

Statistical analysis was performed by using the commercially available statistical SPSS 23.0 software. Comparison of categorical variables was performed by *χ*^2^, and Fisher's exact test was applied when appropriate. Comparison of continuous variables was performed by Mann-Whitney *U* test, and Kruskal-Wallis test was applied when appropriate. Survival analysis was calculated using the Kaplan-Meier method, and differences between the distributions of different groups were assessed by the log-rank test with a 95% confidence level. A Cox proportional hazards analysis was used to determine independent high-risk factors for prognosis. Values of *P* < 0.05 were considered statistically significant.

## 3. Results

### 3.1. Characteristics of Vimentin Expression Levels in EC

Table 1 summarizes the clinicopathologic data of 341 EC patients. The ages in the tables are measured in years and are shown as medians and ranges. Vimentin-positive EC was much more common than vimentin-negative EC (81.23% versus 18.77%, *P* < 0.001). A total of 309 of the 341 cases were type 1 EC, whereas the other 32 cases were type 2 EC, including 10 clear cell carcinomas, 14 serous endometrial adenocarcinomas, 4 mixed serous/endometrioid, and 4 carcinosarcoma endometrial cancers. There was no significant difference between the vimentin-negative and vimentin-positive groups in terms of the frequency of gestation, frequency of parity, age, body mass index (BMI), menopausal status, diabetes, hypertension, hypercholesterolemia, or hypertriglyceridemia. The proportion of type 2 EC in vimentin-negative patients (26.57%) was significantly higher than that in vimentin-positive patients (5.42%). The proportion of vimentin-negative cases (*n* = 17) in type 2 EC was as high as 53.12%, but vimentin-positive cases (*n* = 262) were more common in type 1 EC (84.8%).

### 3.2. Relationships of Vimentin Expression and Other Risk Factors in EC

We found that advanced stages (III and IV) (40.60% versus 14.44%, *P* < 0.001), low differentiation grade (43.75% versus 15.63%, *P* < 0.001), lymph node involvement (25.00% versus 8.30%, *P* < 0.001), lymph vascular space invasion (32.81% versus 16.97%, *P* = 0.006), and deep invasion in the myometrium (46.87% versus 28.88%, *P* = 0.020) prevailed in the vimentin-negative group compared to the vimentin-positive group. We also divided the patients into two groups for stratified analysis according to histological classification. For patients with type 1 EC, the results were almost the same as the whole EC population. Overall, there were significant differences in lymph node involvement, lymph vascular space invasion, myometrium invasion, FIGO stage, and histologic grade between the vimentin-negative and vimentin-positive groups with type 1 EC (Table 2). Conversely, these differences were not found in type 2 EC patients.

### 3.3. Survival Analysis of the Cohort

**Figure 3:**
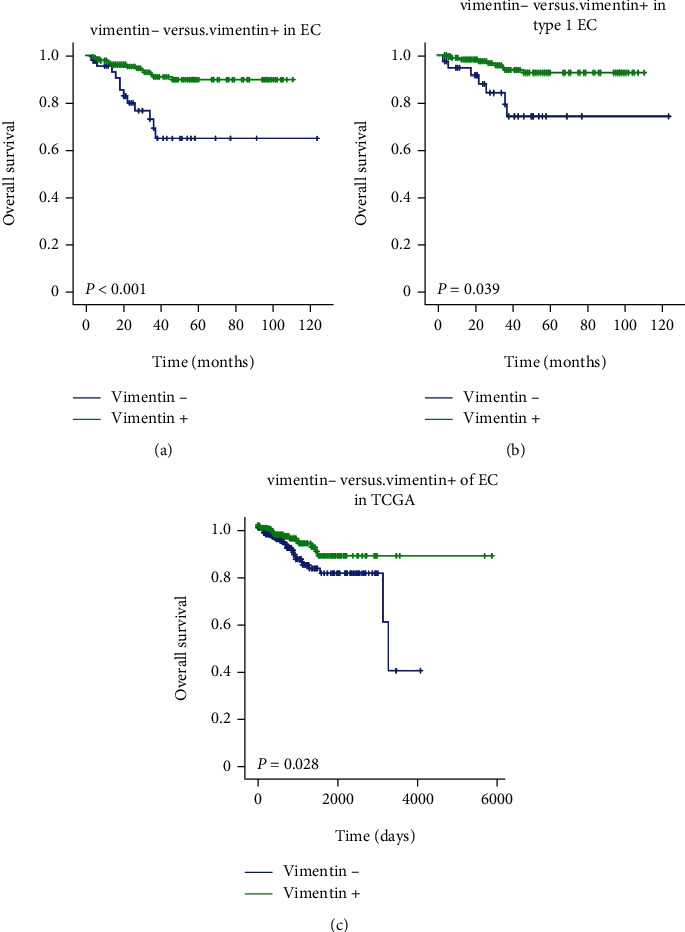
(a) Kaplan-Meier curve estimates the effect of vimentin on overall survival in 341 EC. (b) Kaplan-Meier curve estimates the effect of vimentin on overall survival in 309 tpye1 EC. (c) Kaplan-Meier plot based on VIM mRNA expression shows that the patient group (*n* = 160) with relative low expression levels of VIM has a decreased overall survival rate compared to the patient group (*n* = 250) with a relative high level of VIM expression according to TCGA data.

In this cohort study, KM analysis showed that the OS of vimentin-positive patients was significantly longer than that of vimentin-negative patients in the whole EC population (Figure 3(a)). Lymph node involvement, deep myometrium invasion, high histologic grade, and advanced FIGO stages (III and IV) were all associated with a significantly shorter OS. In stratified analysis, the results were similar as above in type 1 EC (Figure 3(b)), but not in type 2 EC. During a median 22-month follow-up, 69.2% and 91.3% progression-free survival at 3 years were observed in vimentin-negative and vimentin-positive patients, respectively. In Cox univariate analysis, considering the overall survival of the patients, histological type, advanced FIGO stages, high tumor grade, lymph node involvement, lymph vascular space invasion, and deep myometrium invasion were all risk factors (HR: 14.837, 13.327, 7.333, 8.240, 6.539, and 10.360, respectively), whereas overexpression of vimentin was a protective factor (HR: 0.243, 95% CI 0.116~0.512, and *P* < 0.001) (Table 3). In Cox multivariate analysis, histological type, advanced FIGO stages, lymph vascular space invasion, and deep myometrium invasion were independent risk factors, but the significance of vimentin disappeared (Table 3).

### 3.4. Analysis of VIM Gene Expression in TCGA Data

**Figure 4:**
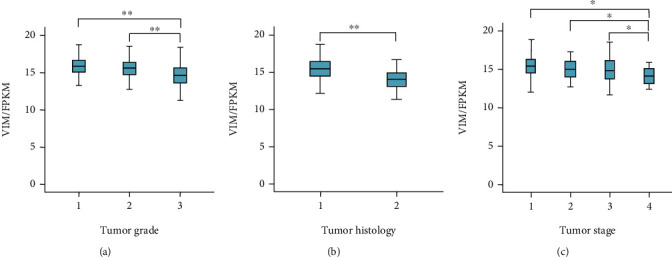
(a) VIM mRNA level decreases as the tumor grade increases. ^∗∗^*P* < 0.001. (b) VIM mRNA level is lower in type 2 EC than in type1 EC. ^∗∗^*P* < 0.001. (c) VIM mRNA level decreases as the tumor stage increases. ^∗^*P* < 0.05.

Bioinformatics analysis of VIM mRNA expression was performed to confirm our experimental results. We utilized gene expression data from the publicly available TCGA data set. The clinicopathological characteristics of 547 EC patients in TCGA are provided in Table 4. To study the association between VIM expression and outcome of patients with EC, VIM gene expression (mRNA) was assessed in 547 EC patients. The mean values of VIM from G1 to G3 were 15.7 FPKM, 15.3 FPKM, and 14.6 FPKM, respectively, with significant differences (*P* < 0.001, Figure 4(a)). The mean values of VIM were 15.3 FPKM in type 1 EC and 13.9 FPKM in type 2 EC, which were also significantly different (Figure 4(b)). Additionally, the mean VIM values for FIGO stages from stage I to stage IV were 15.2 FPKM, 14.7 FPKM, 14.7 FPKM, and 13.4 FPKM, respectively, with significant differences (*P* < 0.001, Figure 4(c)). The level of VIM mRNA expression in moderately and lowly differentiated EC was reduced in comparison with that of highly differentiated EC. In addition, VIM expression in tumors progressively decreased in patients with stage II and III EC compared to those in patients with stage I EC. Overall, these bioinformatics data showed that a decrease in VIM expression correlated with the aggressiveness of EC.

The cutoff value of the low and high VIM expression groups was set at 15.2 FPKM as the median level. Kaplan-Meier analysis based on this cutoff revealed that patients with low VIM expression had a significantly shorter OS than those patients with high VIM expression (*P* = 0.028, Figure 3(c)). However, there was no significant difference in the KM curve in stratified analysis between type 1 and type 2 EC.

### 3.5. GSEA and KEGG Analysis of TCGA Data

**Figure 5:**
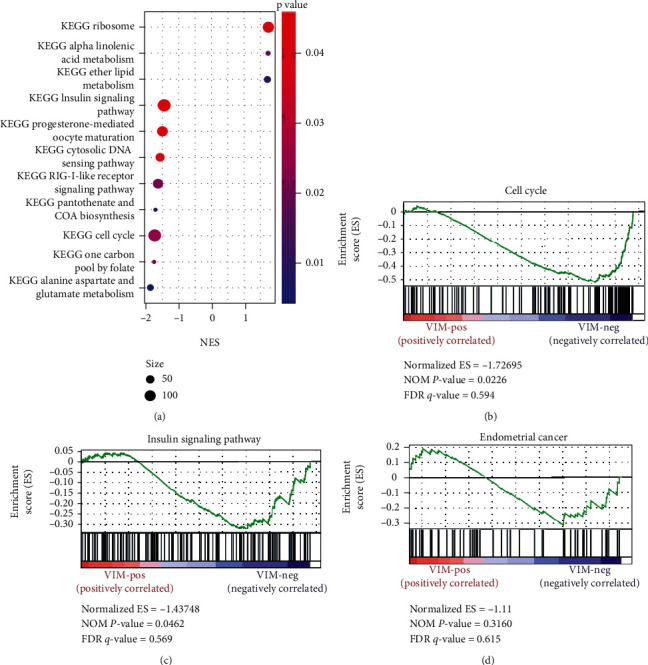
(a) Eleven signaling pathways significantly related to VIM were detected by KEGG analysis. (b) Cell cycle was enriched in VIM low expression phenotype. (c) Insulin signaling was enriched in VIM low expression phenotype. (d) The analysis among VIM and several classical signaling relative to EC did not yield statistically positive results.

Eleven signaling pathways significantly related to VIM were detected by KEGG analysis (Figure 5(a)). However, most of them were associated with metabolism and antiviral infection, and only two pathways were likely to be related to EC (Figures 5(b) and 5(c)).  Figure 5 shows that the cell cycle and insulin signaling were enriched in the VIM-low expression phenotype. Analysis between VIM and several classical signaling pathways related to EC did not yield statistically positive results (Figure 5(d)). By examining the details of pathways, some crossovers between the two pathways and classical pathways in EC, such as MAPK and PI3K signaling, were found, indicating that VIM might be involved in EC development.

## 4. Discussion

Despite advances in medical treatment, there has been little improvement in the 5-year survival rate for EC. Although immunohistochemical panels and molecular indicators may be effective, they are complex and uneconomical for accurately predicting the prognosis of EC. Prognostic factors for EC have always been a hot research topic. As early as 1986, Dabbs et al. described the distribution and role of vimentin in endometrial cancer and cervical adenocarcinoma [20]. They found that coexpression of vimentin and cytokeratin was universally present in normal proliferative endometrial grands, with marked decrease or absence of vimentin staining in secretory phase patterns, and the tumors with clear cell areas and mucinous areas were negative for vimentin but positive for cytokeratin. This is consistent with our findings, which suggests that the role of vimentin in EC may be heterogeneous and distinct from other tumors. We also found that vimentin negativity correlated with lymph node involvement, deep myometrium invasion, lymph vascular space invasion, advanced FIGO stages, and high grade. Negative vimentin expression was more common in type 2 EC than in type 1 EC, and vimentin-negative patients had shorter OS compared to vimentin-positive patients. To our knowledge, this is the first study with such a large cohort and will serve as a basis for future studies.

Vimentin, a 57 kDa protein, is a highly conserved member of the type III intermediate filament protein (IPF) family and is ubiquitously expressed in normal mesenchymal cells. Recent study of Patteson et al. revealed that loss of vimentin enhanced cell motility through small confining spaces [21]. Vimentin can be phosphorylated by Akt1, a kinase activated downstream of Ras and PI3K, and phosphorylation of vimentin protects itself from proteolysis and enhances cell migration and metastasis [22]. Vimentin is overexpressed in many epithelial carcinomas, such as prostate cancer, lung cancer, breast cancer, and gastric cancer, and is associated with tumor invasion and poor prognosis. Recent studies [23, 24] have revealed that vimentin can be translocated to the surface of very aggressive tumor cells, such as metastatic cells. These conclusions are mostly based on clinical data and cell line experiments. Nonetheless, unlike studies on other cancers, our clinical data suggested a better prognosis for vimentin-positive patients with EC. To date, there are limited studies on the relationship between vimentin expression and prognosis of EC. The results of Coppola et al., Papadopoulos et al., and Dabbs et al. confirmed our findings. Vimentin negativity was associated with metastasis of EC. Recent studies have shown that metastasis can occur via mesenchymal–epithelial transition (MET) - dependent and MET-independent routes [25]. Transient EMT and reverse MET rounds are thought to mediate different stages of the metastatic cascade. EMT promotes epithelial tumor cells in situ to disengage, migrate, and enter the blood circulation as well as other organs to form micrometastases. On the other hand, MET allows tumor cells to clone and proliferate into large tumors that have the same properties as tumors in situ [26]. By allowing redifferentiation of disseminated tumor cells, MET may be a crucial process for macrometastasis in many differentiated carcinoma types [27]. Induction of MET may also be a rate-limiting and important step in the metastasis of differentiated primary tumors. Previous studies have detailed the genomic landscape of primary endometrial cancers, but their evolution into metastases has not been characterized. Recurrent metastasis-specific mutations were not significantly discovered [28]. Therefore, we speculate that expression of vimentin is tissue specific that metastasis of EC is heterogeneous, and that MET predominates in EC with large metastases. In contrast to EMT, expression of E-cadherin representing epithelial characteristics increases, while expression of vimentin representing mesenchymal characteristics decreases during MET [29]. The expression level of vimentin in the large metastatic endometrium was low, and a poor prognosis was predicted for the majority of vimentin-negative cases. Sembritzki et al. [30] reported that cytoplasmic wild-type p53 was found in vimentin-positive glioblastoma whereas nuclear p53 was found in vimentin-negative glioblastoma. This suggests that lack of vimentin expression does not allow for its EMT function, and tumor metastasis may be promoted through other routes. Recently, some studies [31–35] have proposed vimentin as a potential molecular target for treatment of some kinds of cancers; however, our results suggest enough prudence in using it for EC.

Our results emphasize the prognostic role of vimentin expression in EC. However, there are some limitations to our study. Our study did not distinguish the primary tumor from the metastatic tumor in the pathological tissue, which may have a slight impact on the results. Our findings showed that low vimentin expression was associated with shortened OS in univariate analysis, but this significant difference disappeared in Cox multivariate analysis. Vimentin appeared to be an independent prognostic factor for EC. This result was also consistent with our correlation analysis, which revealed that vimentin was associated with metastasis, histological classification, FIGO stage, and histologic grade. Vimentin is a protein with diverse and complex functions. Currently, the most commonly used methods of studying the relationships between vimentin and tumors are immunohistochemistry and cell line analyses. Further studies are required to understand the biological implications of reduced vimentin expression and EC aggressiveness. Animal models of EC may be expected to be used to study the role of vimentin in the tumor microenvironment [36].

## 5. Conclusions

In summary, negative vimentin expression correlates with tumor metastasis and worse overall survival. Vimentin may be an excellent prognostic biomarker for EC. The mechanism by which vimentin participates in EC progression needs to be explored.

## Figures and Tables

**Table 1:** Clinicopathological characteristics of the 341 presented EC cases.

Characteristics	*N*	Vimentin negative	Vimentin positive	*P* value
Age (years)	58 (30-93)	60 (37-79)	57 (30-93)	0.017
Gestation	2 (0-8)	2 (0-5)	2 (0-8)	0.614
Parity	1 (0-4)	1 (0-3)	1 (0-4)	0.409
FIGO stage				<0.001
I	250	35 (54.69%)	215 (77.62%)	
II	25	3 (4.68%)	22(7.94%)	
III	52	19 (29.68%)	33 (11.91%)	
IV	14	7 (10.95%)	7 (2.53%)	
Postmenopause				0.921
Yes	214	40 (62.50%)	174 (62.82%)	
No	127	24(37.50%)	103(37.18%)	
Diabetes				0.435
Yes	79	17 (26.56%)	62 (22.38%)	
No	262	47 (73.44%)	215 (77.62%)	
Hypertension				0.289
Yes	163	34 (54.13%)	99 (35.74%)	
No	178	30 (46.87%)	148 (64.26%)	
Hypercholesteremia				0.275
Yes	75	17 (27.42%)	58 (21.40%)	
No	258	45 (72.58%)	213 (78.60%)	
NA	8			
BMI (kg/m^2^)				0.339
<18.5	7	3 (4.76%)	4 (1.45%)	
18.5-23.9	87	17(26.98%)	70 (25.45%)	
24.0-27.9	113	22 (34.92%)	91 (33.09%)	
≥28.0	131	21 (33.34%)	110 (40.01%)	
NA	2			
Hypertriglyceridemia				0.667
Yes	32	5 (8.07%)	27 (10.00%)	
No	230	57 (91.93%)	243 (90.00%)	
NA	9			
Histologic grade				<0.001
1	106	12 (18.75%)	94 (34.18%)	
2	162	24 (37.50%)	138 (50.18%)	
3	39	11 (17.18%)	28 (10.18%)	
High grade	32	17 (26.57%)	15 (5.46%)	
NA	2			
Histological type				<0.001
I	309	47 (73.43%)	262 (94.58%)	
II	32	17 (26.57%)	15 (5.42%)	
Positive lymph nodes				<0.001
Yes	39	16 (25.00%)	23 (8.30%)	
No	302	48 (75.00%)	254 (91.70%)	
LVSI				0.006
Yes	68	21 (32.81%)	47 (16.97%)	
No	219	43 (67.19%)	230 (83.03%)	
MI				0.020
<50%	231	34 (53.13%)	197 (71.12%)	
>50%	110	30 (46.87%)	80 (28.88%)	
Vital state				0.001
Alive	300	50 (79.37%)	250 (94.34%)	
Dead	28	13(20.63%)	15 (5.66%)	
NA	13			

EC: endometrial carcinoma; FIGO: Federation International of Gynecology and Obstetrics Association; BMI: body mass index; NA: NA means that the data is not available; LVSI: lymph vascular space invasion; MI: myometrium invasion. The ages in the table are in years and are shown as medians and ranges.

**Table 2:** Clinicopathological characteristics of the 309 presented type 1 EC cases.

Characteristics	*N*	Vimentin negative	Vimentin positive	*P* value
Age (years)	57 (30-72)	59 (37-79)	57 (30-93)	0.178
Gestation	2 (0-8)	2 (0-5)	2 (0-8)	0.322
Parity	1 (0-4)	1 (0-3)	1 (0-4)	0.911
FIGO stage				<0.001
I	235	26 (55.32%)	209 (79.77%)	
II	25	3 (6.38%)	22 (8.40%)	
III	39	13 (27.66%)	26 (9.92%)	
IV	10	5 (10.64%)	5 (1.91%)	
Postmenopause				0.605
Yes	188	27 (57.45%)	161 (61.45%)	
No	121	20 (42.55%)	101 (38.55%)	
Diabetes				0.253
Yes	72	14 (29.79%)	58 (22.13%)	
No	237	33 (70.21%)	204 (77.86%)	
Hypertension				0.402
Yes	147	25 (53.19%)	122 (46.56%)	
No	162	22 (46.81%)	140 (53.44%)	
Hypercholesteremia				0.624
Yes	69	12 (25.53%)	57 (22.27%)	
No	234	35 (74.47%)	199 (77.73%)	
NA	6			
BMI (kg/m2)				0.079
<18.5	6	3 (6.52%)	3 (1.15%)	
18.5-23.9	77	10 (21.74%)	67 (25.67%)	
24.0-27.9	100	17 (36.96%)	83 (31.89%)	
≥28.0	124	16 (34.78%)	108 (41.49%)	
NA	2			
Hypertriglyceridemia				0.723
Yes	30	4 (8.51%)	26 (10.20%)	
No	272	43 (91.49%)	229(89.80%)	
NA	7			
Histologic grade				0.041
1	106	12 (25.53%)	94 (36.15%)	
2	162	24 (51.06%)	138 (53.08%)	
3	39	11 (23.41%)	28 (10.77%)	
NA	2			
Positive lymph nodes				0.008
Yes	26	9 (19.15%)	17(6.49%)	
No	283	38 (80.85%)	245 (91.51%)	
LVSI				0.007
Yes	54	15 (31.91%)	39(14.89%)	
No	255	32 (68.09%)	223 (85.11%)	
MI				0.241
<50%	221	29 (61.70%)	192(73.28%)	
>50%	88	18 (38.30%)	70 (26.72%)	
Vital state				0.009
Alive	281	40 (85.11%)	241 (96.40%)	
Dead	16	7 (14.89%)	9 (3.60%)	
NA	12			

EC: endometrial carcinoma; FIGO: Federation International of Gynecology and Obstetrics Association; BMI: body mass index; NA: NA means that the data is not available; LVSI: lymph vascular space invasion; MI: myometrium invasion. The ages in the table are in years and are shown as medians and ranges.

**Table 3:** Univariate and multivariate analyses (Cox regression model) of 341 EC.

	Univariate analysis			Multivariate analysis		
	HR	OS 95% CI	*P* value	HR	OS 95% CI	*P* value
Histological type (type 1 vs. 2)	14.837	6.805, 32.350	<0.001	9.935	2.683, 36.790	<0.001
Clinical staging (FIGO I, II vs. III, IV)	13.327	5.856, 30.330	<0.001	8.283	2.993, 22.925	<0.001
Tumor grading (grade 1, 2 vs. 3, high)	7.333	3.445, 15.606	<0.001			
Positive lymph nodes (yes vs. no)	8.24	3.882, 17.490	<0.001			
LVSI (yes vs. no)	6.539	3.088, 13.847	<0.001	2.62	1.043, 6.581	0.040
MI (yes vs. no)	10.36	3.937, 27.259	<0.001	3.771	1.271, 11.188	0.017
Vimentin	0.243	0.116, 0.512	<0.001			

EC: endometrial carcinoma; FIGO: Federation International of Gynecology and Obstetrics Association; LVSI: lymph vascular space invasion; MI: myometrium invasion.

**Table 4:** Clinicopathological characteristics of 547 EC patients in TCGA.

Characteristics	*N*	Vimentin negative	Vimentin positive	*P* value
Age (years)	64 (31-90)	67 (33-90)	61 (31-89)	<0.001
FIGO stage				<0.001
I	342	152 (55.27%)	190 (69.85%)	
II	52	28 (10.18%)	24 (8.82%)	
III	123	70 (25.45%)	53 (19.49%)	
IV	30	25 (9.09%)	5 (1.84%)	
Menopause				<0.001
Premenopause	35	6 (2.36%)	29 (11.03%)	
Perimenopause	17	8 (3.15%)	9 (3.42%)	
Postmenopause	448	234 (92.13%)	214 (81.37%)	
Indeterminate	17	6 (2.36%)	11 (4.18%)	
BMI (kg/m^2^)				0.262
<18.5	5	3 (1.16%)	2 (0.72%)	
18.5-24.9	110	53 (20.54%)	57 (20.43%)	
25.0-29.9	115	57 (22.09%)	58 (20.79%)	
≥30	307	145 (56.20%)	162 (58.06%)	
NA	30			
Histologic grade				<0.001
1	99	27 (9.82%)	72 (26.47%)	
2	122	38 (13.82%)	84 (30.88%)	
3	315	199 (72.36%)	116 (42.65%)	
High grade	11	11 (4.00%)	0 (0.00%)	
Residual tumor				0.059
R0	376	181 (79.39%)	195 (85.90%)	
R1	22	15 (6.58%)	7 (3.08%)	
R2	16	12 (5.26%)	4 (1.76%)	
RX	41	20 (8.77%)	21 (9.25%)	
NA	92			
Histological type				0.002
I	410	160 (58.18%)	250 (91.91%)	
II	137	115 (41.82%)	22 (8.09%)	
Vital state				0.047
Alive	502	246 (89.45%)	256 (94.12%)	
Dead	45	29 (10.55%)	16 (5.88%)	

EC: endometrial carcinoma; TCGA: The Cancer Genome Atlas; FIGO: Federation International of Gynecology and Obstetrics Association; BMI: body mass index; NA: NA means that the data is not available; RX: RX meant residual tumor in stages more than 2. The ages in the table are in years and are shown as medians and ranges.

## Data Availability

The demographic data and clinical information data used to support the findings of this study are available from the corresponding author upon request. The Cancer Genome Atlas (TCGA) data was collected from Genomic Data Commons (GDC) (https://portal.gdc.cancer.gov/).

## References

[1] Goebel E. A., Vidal A., Matias-Guiu X., Blake Gilks C. (2018). The evolution of endometrial carcinoma classification through application of immunohistochemistry and molecular diagnostics: past, present and future. *Virchows Archiv*.

[2] Sung H., Ferlay J., Siegel R. L. (2021). Global cancer statistics 2020: GLOBOCAN estimates of incidence and mortality worldwide for 36 cancers in 185 countries. *CA: a Cancer Journal for Clinicians*.

[3] Amant F., Moerman P., Neven P., Timmerman D., van Limbergen E., Vergote I. (2005). Endometrial cancer. *The Lancet*.

[4] Lu K. H., Broaddus R. R. (2020). Endometrial cancer. *The New England Journal of Medicine*.

[5] Travaglino A., Raffone A., Mascolo M. (2020). TCGA molecular subgroups in endometrial undifferentiated/dedifferentiated carcinoma. *Pathology Oncology Research*.

[6] Danielsson F., Peterson M. K., Caldeira Araújo H., Lautenschläger F., Gad A. K. B. (2018). Vimentin diversity in health and disease. *Cell*.

[7] Ivaska J. (2011). Vimentin: central hub in EMT induction?. *Small GTPases*.

[8] Mendez M. G., Kojima S., Goldman R. D. (2010). Vimentin induces changes in cell shape, motility, and adhesion during the epithelial to mesenchymal transition. *The FASEB Journal*.

[9] Odero-Marah V., Hawsawi O., Henderson V., Sweeney J. (2018). Epithelial-mesenchymal transition (EMT) and prostate cancer. *Advances in Experimental Medicine and Biology*.

[10] Sharma P., Alsharif S., Fallatah A., Chung B. M. (2019). Intermediate filaments as effectors of cancer development and metastasis: a focus on keratins, vimentin, and nestin. *Cell*.

[11] Yin S., Chen F. F., Yang G. F. (2018). Vimentin immunohistochemical expression as a prognostic factor in gastric cancer: a meta-analysis. *Pathology, Research and Practice*.

[12] Domagala W., Striker G., Szadowska A., Dukowicz A., Harezga B., Osborn M. (1994). P53 protein and vimentin in invasive ductal NOS- breast carcinoma-relationship with survivaland sites of metastases. *European Journal of Cancer*.

[13] Coppola D., Fu L., Nicosia S. V., Kounelis S., Jones M. (1998). Prognostic significance of p53, bcl-2, vimentin, and S100 protein-positive Langerhans cells in endometrial carcinoma. *Human Pathology*.

[14] Papadopoulos N., Kotini A., Cheva A. (2002). Immunohistochemical expression of vimentin and secretory component antigens in endometrial hyperplasia and neoplasia. *European Journal of Gynaecological Oncology*.

[15] Nesina I. P., Iurchenko N. P., Buchynska L. G. (2018). Markers of the epithelial-mesenchymal transition in cells of endometrial carcinoma. *Experimental Oncology*.

[16] Satelli A., Li S. (2011). Vimentin in cancer and its potential as a molecular target for cancer therapy. *Cellular and Molecular Life Sciences*.

[17] Jones H. W. (1999). The importance of grading in endometrial cancer. *Gynecologic Oncology*.

[18] Liu C. H., Jiang Q. P., Lin D. (2016). Coexpression of MAP2K4 and vimentin proteins in human endometrial carcinoma and its clinicopathological significance. *Nan Fang Yi Ke Da Xue Xue Bao*.

[19] Shi Z. G., Li S. Q., Li Z. J., Zhu X. J., Xu P., Liu G. (2015). Expression of vimentin and survivin in clear cell renal cell carcinoma and correlation with p53. *Clinical & Translational Oncology*.

[20] Dabbs D. J., Geisinger K. R., Norris H. T. (1986). Intermediate filaments in endometrial and endocervical carcinomas: the diagnostic utility of vimentin patterns. *The American Journal of Surgical Pathology*.

[21] Patteson A. E., Pogoda K., Byfield F. J. (2019). Loss of vimentin enhances cell motility through small confining spaces. *Small*.

[22] Zhu Q. S., Rosenblatt K., Huang K. L. (2011). Vimentin is a novel AKT1 target mediating motility and invasion. *Oncogene*.

[23] Batth I. S., Li S. (2020). Discovery of cell-surface vimentin (CSV) as a sarcoma target and development of CSV-targeted IL12 immune therapy. *Advances in Experimental Medicine and Biology*.

[24] Patteson A. E., Vahabikashi A., Goldman R. D., Janmey P. A. (2020). Mechanical and non-mechanical functions of filamentous and non-filamentous vimentin. *BioEssays*.

[25] Somarelli J. A., Schaeffer D., Marengo M. S. (2016). Distinct routes to metastasis: plasticity-dependent and plasticity-independent pathways. *Oncogene*.

[26] Chaffer C. L., Brennan J. P., Slavin J. L., Blick T., Thompson E. W., Williams E. D. (2006). Mesenchymal-to-epithelial transition facilitates bladder cancer metastasis: role of fibroblast growth factor receptor-2. *Cancer Research*.

[27] Brabletz T. (2012). To differentiate or not--routes towards metastasis. *Nature Reviews. Cancer*.

[28] Gibson W. J., Hoivik E. A., Halle M. K. (2016). The genomic landscape and evolution of endometrial carcinoma progression and abdominopelvic metastasis. *Nature Genetics*.

[29] Serrano-Gomez S. J., Maziveyi M., Alahari S. K. (2016). Regulation of epithelial-mesenchymal transition through epigenetic and post-translational modifications. *Molecular Cancer*.

[30] Sembritzki O., Hagel C., Lamszus K., Deppert W., Bohn W. (2002). Cytoplasmic localization of wild-type p53 inglioblastomas correlates with expression ofvimentin and glial brillary acidic protein. *Neuro-Oncology*.

[31] Ohara M., Ohara K., Kumai T. (2020). Phosphorylated vimentin as an immunotherapeutic target against metastatic colorectal cancer. *Cancer Immunology, Immunotherapy*.

[32] Yang H., Li X., Meng Q. (2020). CircPTK2 (hsa_circ_0005273) as a novel therapeutic target for metastatic colorectal cancer. *Molecular Cancer*.

[33] Wang W., Yi M., Zhang R. (2018). Vimentin is a crucial target for anti-metastasis therapy of nasopharyngeal carcinoma. *Molecular and Cellular Biochemistry*.

[34] Trogden K. P., Battaglia R. A., Kabiraj P., Madden V. J., Herrmann H., Snider N. T. (2018). An image-based small-molecule screen identifies vimentin as a pharmacologically relevant target of simvastatin in cancer cells. *The FASEB Journal*.

[35] Tian H., Lian R., Li Y. (2020). AKT-induced lncRNA VAL promotes EMT-independent metastasis through diminishing Trim16-dependent Vimentin degradation. *Nature Communications*.

[36] Moiola C. P., Lopez-Gil C., Cabrera S. (2018). Patient-derived xenograft models for endometrial cancer research. *International Journal of Molecular Sciences*.

